# Vision Testing in Late Nineteenth- and Early Twentieth-Century Britain: Opticians, Medical Practitioners and the Battle for Professional Authority

**DOI:** 10.1093/shm/hkab122

**Published:** 2021-11-26

**Authors:** Gemma Almond

**Keywords:** opticians, professionalisation, medical authority, medicalisation, retail

## Abstract

In the 1890s, opticians were reforming their practice against a body of medical practitioners who were increasingly attempting to specialise in, and monopolise, vision testing and spectacle dispensing. This article explores how and why vision testing became a subject of debate and how opticians were able to successfully set out their claims to professional authority in the face of medical competition. It argues that opticians created a scientific rhetoric distinctive from medical training by combining optics and anatomy. In response, medical practitioners attempted to consolidate the medicalisation of an area of the body through claiming completely new, and potentially unfounded, areas of expertise and medical jurisdiction. A study of the optician’s role in the 1890s demonstrates the broader influence of fringe professions, commercial marketing and the public’s receptiveness to the construction of expertise in enabling but ultimately inhibiting the medicalisation process, an outcome that medical practitioners had to grudgingly accept.

On 21 April 1898 *The Optician* reported on a recent court meeting held by the Spectacle Makers’ Company. The meeting included a lecture by Dr George Lindsay Johnson, a Fellow of the Royal College of Surgeons, and was attended by the company’s members and the special committee that had been appointed to deal with the certification of opticians. Johnson was a vocal supporter of the education and certification of opticians. In this meeting he was supported by Ernest Clarke, surgeon to the central London Ophthalmic Hospital and Miller Hospital, who believed that opticians’ certification would aid medical practice. However, the certification of opticians was set against a backdrop of concern over professional boundaries. In this meeting both Johnson and Clarke emphasised their desire for peace, believing that co-operation could be achieved through clearly dividing the roles of the ophthalmic surgeon and the optician based on two definitive skillsets: physiology and physics. While the ophthalmic surgeon had received medical training, Clarke argued that they ‘could no more make a pair of spectacles than fly’.^1^

By drawing upon the broader theme of expanding medical authority in the nineteenth century and the processes of medical specialisation, medicalisation and professionalisation, this article explores two primary questions: why did vision aid dispensing become a subject of debate in the 1890s? and how did opticians set out their claims to professional authority in the face of medical competition? Historicising the selling and dispensing of spectacles, and those involved, is complex. Spectacles and the treatment of eye diseases had been a lucrative commercial market for centuries. Spectacles featured in the stocklists of a range of high street stores and in the pockets of a variety of street sellers. Simultaneously, their sale featured in the practice of an ‘oculist’, a term that could denote a fraudulent quack advertising ‘cures’ blindness and eye disease as well as an emerging specialist with medical training. By the 1890s the terms ‘ophthalmic surgeon’, ‘ophthalmologist’ and ‘oculist’ were used interchangeably in the discussions between medical practitioners and opticians to denote a specialism in the diseases of the eye. The newly titled ‘ophthalmic surgeon’ aligned the older term ‘oculist’ with the professional emergence of ophthalmology in the nineteenth century, thus helping to give it an air of authority and create a more respectable title. The terms oculist and ophthalmologist were used interchangeably by some contemporaries and feature in this article through their use as pseudonyms. However, I will use the term ophthalmic surgeon when discussing the medical practitioners that specialised in the eye and became involved in the debates because practitioners more readily identified themselves as such in the medical texts that they produced. Indeed, the use of the term ‘ophthalmic surgeons’ by these practitioners was indicative of the position and status that they were attempting to reinforce.

The public recognised opticians, and not ophthalmic surgeons, as spectacle dispensers in the nineteenth century and turned to the high street to alleviate their declining vision. In the first half of the century, ophthalmic surgeons would have been inclined to agree and considered spectacle dispensing ‘beneath’ their attention.^2^ At the beginning of the nineteenth century, vision ‘testing’ consisted of a person trying on and ultimately choosing their spectacles under the guidance of the retailer. However, the work of F.C. Donders on the refractive and accommodative condition of the eye in 1864—which utilised the ophthalmoscope that had been invented by Hermann von Helmholtz in 1851—altered this medical stance. Ophthalmologists medicalised refractive vision errors and rooted them in understandings of physiology and pathology. Certain conditions, such as myopia, became defined as a disease that required treatment or cure.

These new jurisdictional claims by ophthalmologists formed part of the medicalisation process. In the case of refractive vision errors, ophthalmic surgeons argued that spectacles should be prescribed by medically trained personnel because the work of Donders and Helmholtz had made the vision test more complex. By the second half of the nineteenth century, a person’s vision was tested using optotypes—the sight test chart—and incorporated diagnostic equipment to confirm test outcomes. In addition, the adoption of the dioptric as the universal lens measurement in 1878, further increased the precision required. The ability to objectively measure the refractive condition of a person’s eye, and the greater number of lens gradations available, meant the dispenser had a more significant role in performing the test and identifying the appropriate lens type and strength. The use of sophisticated techniques was not initially universal and both opticians and medical practitioners interchangeably performed the older-style and new, more complex, test. Nevertheless, medical practitioners utilised technology and physiological knowledge to legitimise their stance that vision testing should be conducted only by medically trained personnel.

The meaning and role of an optician had to evolve in response to changes to the vision test. Earlier use of the term had denoted a dealer in optical goods or a person with knowledge of optics and the construction of optical instruments. The shift from geometrical to physiological optics, however, led some opticians from the 1860s to adopt embellishments that alluded to a new form of expertise, such as ‘ophthalmic optician’ and ‘oculist optician’. The retailing optician in the 1890s varied. It could signify a specialist in optical, mathematical or philosophical goods, be an additional term used in a business name by chemists, jewellers, watchmakers and stationers to signify they sold spectacles, *or* a term used to identify an individual that studied, and specialised in, the measurement of vision and the treatment of simple refractive conditions. This diversity illuminates the early development of two distinct groups, the optician that fitted and constructed frames and an early form of an optometrist, specialising in vision testing and spectacle prescribing. The debates and conflict between these groups of traders—those that proclaimed physiological knowledge and those that did not—reveal the mechanism of opticians’ professionalisation. Opticians perceived vision testing and an expertise beyond the selling of optical goods paramount to achieving a professional identity.

The intensity of the debate among ophthalmologists and opticians reflects the professional implications of gaining a monopoly on vision testing. The medical practitioner and optician sought to regulate spectacle selling while being acutely aware of its commercial potential and the number of prospective Victorian consumers. Victorian societal and environmental changes had levied the importance of vision. Cities, street signs and house numbers demanded an unprecedented level of visual acuity.^3^ In addition, as shown in Peter John Brownlee’s more recent study, new markets in America, such as the expanding production of print and visual culture, depended upon the ‘economy of the eyes’.^4^ In Britain as well as America, debates between opticians and medical practitioners emerged against a backdrop of visual acuity, visual attentiveness and colour perception increasingly becoming part of state and national discussions of national efficiency and the need to manage, measure and rationalise visual perception.^5^ In the context of the bureaucratisation of the senses, then, ophthalmologists aspired to position themselves as the public expert, able to offer a specific body of knowledge that could be used to measure the efficiency of the workplace and the overall ocular health of the British nation.

While Caroline Weaver has argued that the British state’s involvement in vision and vision testing intensified in the 1920s, a study of the 1890s allows the actions of ophthalmologists and opticians in the early twentieth century to be more fully understood.^6^ The debates between opticians and ophthalmologists emerged in response to ophthalmologists using the changing nature of the vision test to further their attempts to consolidate their professional identity as a medical specialism. In Britain, medical specialisation did not readily gain acceptance and practitioners were slower to identify its benefits for medical research and clinical science. In contrast to America, Germany and France, mistrust towards specialisation in Britain created a much less visible and straightforward form of acceptance by the end of the nineteenth century.^7^ While ophthalmology was at the forefront of studies of medical specialisation in Britain, the ophthalmic surgeon in vision testing debates had only emerged as part of a more cohesive body of practitioners in the second half of the century with the establishment of the Ophthalmological Society in 1880.^8^ In 1881 the president of the Ophthalmology Section at the British Medical Association reflected on the broader international community of ophthalmologists and how the medical world viewed their work to be ‘of the highest value’ to the ‘whole field of medicine’. It highlights how ophthalmic surgeons, along with other specialists, principally concerned themselves with the legitimacy of their practice and both medical and popular recognition of their position and status. Ophthalmologists and ophthalmic surgeons therefore drew upon diagnostic equipment, the rhetoric of science and the concept of ‘precision’ to develop their professional standing as specialists.^9^ The new and ill-defined role of vision testing in the general medical and ophthalmological curriculum, however, weakened the legitimacy of their position against opticians who could similarly claim technological expertise and a much longer tradition of vision testing expertise.

A study of ophthalmic surgeon’s actions in the 1890s therefore contributes to our understanding of the ways in which medical practitioners attempted to consolidate and broaden their professional authority in the nineteenth century. A number of historians have analysed the methods used by practitioners to either subjugate or completely remove occupations that straddled the borders of their practice, including nursing, pharmaceuticals, dentistry and orthodontics.^10^ Drawing upon the rhetoric of science, alternative groups, such as pharmacists in nineteenth-century Canada, were able to use the same or similar techniques as medical practitioners in order to develop a distinct, credible, professional identity.^11^ The optician becomes an important and crucial example for exploring the methods used by bordering groups to create a professional identity and define the limits of nineteenth-century medical authority and medical specialism. Opticians used their scientific knowledge and understanding of optics to justify their position against the medical curriculum. Indeed, the optician was not passive to broader changes in medical thought and practice. Individually, they responded by publishing their own texts on the anatomy and testing of the eye, setting up vision testing rooms on their premises, and altering their roles so as to cater for, and accommodate, increasing demand. Additionally, opticians straddled the boundaries of theoretical knowledge and trade. Although, as has been more recently argued, some practitioners were engaged in more commercial practices and this was not necessarily a barrier to professional identity, the duality of an opticians’ position provides an interesting case-study for exploring the role of commercialism in the formation of British professional identities.^12^ It reveals the processes by which medical practitioners attempted to consolidate the medicalisation of an area of the body through claiming completely new, and potentially unfounded, areas of expertise and medical jurisdiction in a bid to obtain commercial monopoly. As argued by S.E.D. Shortt in his study of nineteenth-century medical practitioners, claims of expertise did not necessarily mean that the practice or the application of knowledge of science and medicine actually improved.^13^ In this case, claims by both opticians and ophthalmic surgeons reveal how public reception of ideas—not necessarily practice—determined the success of establishing a profession’s area of expertise.

This article argues that opticians’ attempts to create a more established professional identity led to conflict over the practise of vision testing and vision aid dispensing in the 1890s and shaped the nature of the debates that would continue throughout the first half of the twentieth century. My argument is structured around the implementation of examinations and the certification of opticians from 1897. The first section explores the content of, and correspondence in, *The Optician* between 1891 and 1897. In these years, *The Optician* journal became closely linked to, and reported on, two examining bodies that emerged in the 1890s: the newly established British Optical Association (hereafter BOA) and the Spectacle Makers Company (hereafter SMC), founded in 1629. The second section then focuses on the effects of examinations and certification in *The Optician* and *British Medical Journal* between 1897 and 1904, the end of our period of study and when the SMC finally included vision testing in its examination. It assesses how certification amplified tensions and limited the success of medical practitioners’ response. While histories of both the BOA and SMC exist and acknowledge medical opposition, their institutional focus has limited their ability to fully assess a key component of the professionalisation process: how or why medical opposition to opticians’ professionalisation arose.^14^ This article addresses this lacuna by drawing upon the inter-professional debates in *The Optician* and *British Medical Journal. The Optician* and *British Medical Journal* are fundamental in exploring the relationship between opticians and the medical profession. The two publications emerged as the public voice of their respective professions and their articles and correspondence detail the conflicts that occurred and the reasons behind them.

The debates between opticians and ophthalmic surgeons reveal that inter-professional competition both promoted and limited medicalisation and ultimately hindered the establishment of vision testing as part of ophthalmology’s jurisdiction of professional expertise. Opticians drew upon medical definitions and their medical knowledge to justify their position. However, equally important, was the scientific knowledge required to produce and understand the use of lenses and optics as a treatment method. Certification amplified tensions and limited the success of medical practitioners’ response because it helped opticians successfully maintain their position as vision aid dispensers amongst the general public. The ability of opticians to do so highlights how patients, or customers, can be active collaborators in limiting the extent of medicalisation by not seeing a condition as a ‘medical problem’.^15^ Yet the focus on the methods and knowledge of vision testing shows that opticians drew upon medical definitions and medical knowledge to develop their public image. Thus, an external profession with knowledge of both science and medicine did not fully hinder the process of medicalisation but limited the extent of medical authority and medical practitioners’ ability to control or subjugate a trade—a position that medical practitioners had to grudgingly accept.

## Establishing a Battleground: *The Optician*, Medical Practitioners and Reform, 1891–1897

Changes in vision aid testing altered the sites of vision aid prescribing and dispensing. On the one hand, increased medical knowledge of the eye had laid greater emphasis on the need for a subjective vision test by trained personnel. This transformed how traders advertised, designed their premises and performed vision tests, and also helped to establish the hospital as an alternative site for vision aid prescribing.^16^ On the other hand, demands for a more complex test exposed the lack of regulation at a time when the increasing demand for, and marketability of, spectacles and eyeglasses had expanded the number of retailers involved in vision aid prescribing and dispensing by the 1890s. Changes in vision testing therefore created a competing space for vision aid prescribing based on a set of two distinct practices: medical practitioners in the hospital with medical training and traders often having served an apprenticeship in a trade relating to optics, scientific instruments or metalwork.^17^ These changes did not create a straightforward hierarchy of expertise; not all medical practitioners had been trained in vision testing and not all traders were completely unqualified. Indeed, *The Optician* journal in its opening articles acknowledged the variability in vision aid dispensing and prescribing practices. First, it understood that medical involvement had increased but that sufficient changes to the medical curriculum had yet to take place. Second, it recognised their own need to professionalise and address the diverse number of traders becoming involved.

Historians’ use of the term ‘professionalisation’ is vague but has been more broadly defined as the creation of a distinct occupational group with a particular knowledge base.^18^ Within this definition, journals, education, certification, a society or organisation, and a code of ethics have been considered markers of professionalism. Andrew Abbot’s influential study, however, has demonstrated that focusing on the organisation and form of professions masks the true nature of a profession; professions are interdependent and controlled, even defined, by jurisdictional boundaries. Abbott argues that a profession emerges when there is a vacancy and/or a profession seeks to enlarge its own jurisdiction.^19^ In this context, changes in the nature of the vision test created a vacancy and a jurisdictional dispute over control. More recently, inter- and intra-professional competition for authority has also been located at the heart of the medicalisation process.^20^ Opticians therefore established *The Optician* journal in 1891 to promote professionalisation and respond to both internal and external conflict. Similar to other competing groups or occupations in the nineteenth century, opticians utilised their journal for improving communication, education and the likelihood of implementing a system of certification and education to combat medical encroachment.^21^ Parallels, for example, can be drawn between the centrality of journals in the promotion of professional identity—such as *The Lancet* or *Forceps—*and the role of *The Optician* in the creation of a ‘profession’ of vision-aid dispensers.^22^

Launched on 2 April 1891, *The Optician* called for greater knowledge and a more standardised, cohesive community. In its leading article the journal argued that ‘the consequent returns for this are fairly manifest, and the day will be welcomed by all when the covert sneer contained in the epitaph “Shoptician” shall have been, once and for all, wiped out’.^23^ Opticians’ aspirations to achieve professional status formed part of a broader move amongst nineteenth century retailers to achieve a professional or reputable identity over less credible traders.^24^ Opticians used similar techniques of patronage, claims of experience and personal testimonies that emerged in the eighteenth century to distinguish retailers from the unqualified.^25^ Akin to the retail of a host of corrective body technologies that had emerged in the eighteenth century, opticians also increasingly advertised and sought endorsements from medical practitioners or institutions.^26^ However, here, the uniqueness of opticians’ practices is exposed. Unlike the sale of other body technologies, opticians did not immediately seek medical endorsements and references to medical practice or knowledge appear much later and only in substantial quantities in the second half of the nineteenth century following changes to the vision test. ‘The Certificated Optician’, a leading header in the journal on 20 August 1891, was a response to new vision testing methodologies and the encroachment of ophthalmic surgeons.

The certificated or certified optician was well supported in correspondence.^27^ Opticians writing in the journal considered an institute of certified opticians to be the best means of tackling the lack of regulation. Indeed, a centralised system of education and distinctions between the qualified and unqualified have been considered necessary for establishing other professional communities in the nineteenth century more generally.^28^ While an optical institute was mentioned as early as 1891, it received focused attention from 1893. On 21 September, the journal proposed that ‘An Institute of Qualified Opticians’ would ‘purge’ reputable opticians from the ‘irresponsible quacks’, which sparked months of correspondence about the logistics, benefits and challenges of an institute for the advancement of an optician’s status.^29^ However, while there was a general consensus that an institute and regulation would be beneficial, it was initially unclear what the qualifications of an optician should be. The claim that ‘a spectacle vendor is not an Optician’ in correspondence from September 1891 highlights the early developments of a group of optometrists, a body of opticians that specialised in vision testing.^30^ Yet this reshaping of an opticians’ role was not unanimously shared, which made identifying an optician’s specific role and expertise difficult, even amongst opticians themselves. Writing in 1891 a correspondent considered a ‘*competent* optician’ to have knowledge of the ‘laws of light’, the ‘construction of instruments’, and the ‘physical and chemical properties of the materials he employs.^31^ In contrast, correspondence in early 1892 on the technical education of opticians focused on whether opticians would be appropriately trained in vision testing and the appropriate fitting of spectacles to a person’s face.^32^

Exploring the information provided in the journal’s pages highlights the duality of an optician’s role in the 1890s. Not surprisingly, it included a range of scientific and optical information that it thought would benefit its readers.^33^ Yet it also ran standalone articles on the physiology of the eye and several topics associated with the new and emerging vision testing techniques. Similar to dentistry, retaining and acknowledging a connection with medicine was integral to the journal’s commitment to elevating the trade’s status.^34^ The quantity of information is equally revealing; it reflects the journal’s role as a pedagogical tool to encourage opticians to ‘upskill’ to meet the new demands of the vision test. A number of innovative technologies for determining the refractive errors of the eye were discussed.^35^ Articles also reviewed various methodologies for vision testing, including astigmatism and general information on determining the eye’s refractive index.^36^ However, in a bid to ensure that opticians received the fundamentals of optical practice, the journal established ‘The Optical Dictionary’, ‘The Optician’s Library’ and ‘Papers for Beginners’.^37^ These three resources intended to provide preliminary education and act as a reference source for basic terms and key texts, which also encompassed new medical publications. Discussions of instruments, for example, could be informed by medical practitioners, including a text on the ophthalmoscope that was serialised in several issues in 1895.^38^ A number of papers given by prominent ophthalmologists at the meetings of the British Medical Association and the Ophthalmological Society were also published or summarised, guiding opticians to relevant medical texts.^39^ Topics could vary from the relationship between myopia and ‘intellectual progress’ in 1891 to the influence of occupations and substances such as tobacco on the eyesight in 1895.^40^ The discussion of these papers highlights how opticians drew upon the latest research to educate and position themselves as experts in the socio-industrial changes that concerned the state in relation to the condition, and preservation, of the nation’s ocular health.

The focus on vision testing within the journal emphasised the centrality of scientific and medical knowledge to *The Optician’s* professionalising aim. The journal’s stance on vision testing, however, created internal and external medical tensions that subjected opticians’ practice to greater scrutiny. While the debates that emerge between medical practitioners and opticians in the journal are largely anonymous—and therefore tell us little about the distribution, number or type of trader and/or practitioner that supported them—their pseudonyms and choice of language in their letters are able to clearly show the rhetoric that each body was drawing upon and their attempts to consolidate distinctive roles. Medical practitioners did recognise the importance of regulation in the early 1890s. However, the medical profession’s opinion on the optician’s role within this regulation conflicted with the ideas presented by several opticians in the journal. A debate over ‘fair play’ that emerged between April and July 1891 reveals how questions about surgeons’ monopoly, favouritism and power by opticians quickly evolved into a medical response that questioned the dispensing practices of opticians in general.^41^ An ‘oculist’, responding to opticians’ accusations of ophthalmic surgeon’s favouritism, for example, argued: ‘what does your correspondent think would be the percentage of lenses found with dealers in spectacles, that would stand the test of the inspector?’ The ‘oculist’ did not consider all opticians to be deficient and did allude to being confident of certain ‘specified opticians’ abilities.^42^ However, the role and status of the ‘specified opticians’ who were able to meet medical practitioner’s approval was not static in the 1890s and was increasingly narrowly defined as an optician that did not claim any vision testing expertise. Early disapproval of opticians’ attempts to improve their education or standing by medical practitioners is evident in ophthalmic surgeons’ successful attempts to block proposals for technical education by the SMC in 1892.^43^ While *The Optician* quickly attempted to alleviate tensions by publishing a statement stating that they were not in ‘active conflict’ with ‘oculists’, the ‘obstacles’ that ‘oculists’ placed in the way of SMC training revealed that there was a discrepancy between their views on the optician’s appropriate role.^44^

Tensions between opticians and medical practitioners stemmed from a lack of clear professional boundaries. Importantly, medical practitioners as well as opticians centred their discussion of their respective roles on the way in which vision testing and understanding of the physiology of the eye had changed. On 4 January 1894, another medical practitioner writing under the pseudonym ‘Surgeon Oculist’ sparked 2 months’ worth of correspondence in *The Optician*. ‘Surgeon Oculist’ argued that there were many ‘fatal objections’ to the proposal of the certification of opticians if they were to be examined on their ability to recognise diseases of the eye when testing vision. Without medical training, the ‘Surgeon Oculist’ ‘emphatically asserted’ that the optician would not be ‘capable of detecting the fine border-line often separating disease and errors of refraction’.^45^ The ‘Surgeon Oculist’ rooted medical practitioners’ objection to certification in opticians’ attempts to claim physiological and anatomical knowledge of the eye. This did provoke responses by opticians that criticised the professional jealousy, selfishness and the ‘unjust monopoly’ of medical practitioners.^46^ However, there were also justifications of opticians’ practice and their ability to detect abnormalities and refer patients when necessary.^47^ The intensity of these discussions is reflected in the language used by opticians. A Twickenham optician, for example, argued that the ‘Surgeon Oculist’ had ‘sounded the trumpet of hostility’ and had seen ‘war proclaimed by this letter’.^48^ In response the ‘Surgeon Oculist’ stated that it was not a matter of professional jealousy and was instead about securing the welfare of the general public. The ‘Surgeon Oculist’ acknowledged that the endeavour of *The Optician* to elevate the trade to a profession was an ‘honest’ one if the opticians involved were not ‘meddling’ in areas that required ‘special anatomical and medical knowledge and experience’.^49^ This debate revealed that opticians believed themselves to be capable of testing vision and recognising complicated cases, while medical practitioners believed that the appropriate role of the optician was to receive the prescriptions that had been obtained from an eye examination and vision test conducted by an ophthalmic surgeon.

Opticians justified their role against increasing medical criticism in the 1894 debates by arguing that the ‘oculist’s’ skill was ‘dependent on the skill of the optician’ for providing accurate lenses and also for providing a service for the ‘many’ who could not afford an ‘oculist’s’ fee.^50^ Opticians used their independent skillset to promote their own expertise and challenge medical authority. The editors of *The Optician* in early 1895 devoted space for publishing their opinion on the purpose and qualifications of the optician in relation to the ophthalmic surgeon. The journal argued that

Ophthalmic physicians might like to fit spectacles if they could. But, as they never happen to possess the requisite mechanical skill, that ends the matter… The serious study of optics has no part in the curricula of our medical schools… The medical and surgical treatment of *diseases* of the eye is, of course, a matter with which no medically unqualified and self-respecting Optician would care to meddle. But the diagnosis of refraction, and their correction, is, we repeat equally foreign to the practice of medicine and surgery.^51^

A week later the journal further argued that their ‘science of geometrical optics is *entirely* distinct’ from medical training.^52^ The journal concluded its argument in the following week by stating that it was ‘absurd’ that the ‘virtue of what is now understood as medical training’ is enough to prescribe vision aids and medical practitioners should not ‘arrogate themselves’.^53^ The journal sought to demonstrate that opticians were ‘equally as intelligent’ and fundamentally co-equal.^54^ Thus, *The Optician* was attempting to establish a professional boundary that was based on an increasingly defined and credible skillset, which included mechanical skill and knowledge of physics in order to examine and treat the refractive state of the eye. While medical practitioners attempted to assume a superior position, opticians and certain medical practitioners were quick to point out the lack of optical training in the medical curriculum.^55^ Theoretical knowledge of optics and refraction did appear in some ophthalmology textbooks from the late 1880s; however, their authors were still required to impress the importance of the topic in their prefaces and faced the challenge of legitimising specialist training.^56^ A text written primarily for students beginning their study of ophthalmology in 1895, for example, had less emphasis on the understanding of optics and refractive vision errors in their section on eye examination.^57^

There is some evidence of a positive dialogue between the two bodies based on public receptiveness. In March 1896 an article on ‘Can Opticians be Trusted to Correct Defects of Sight? By an Oculist’ in *The Optician* supported some of the claims that had been made by opticians by stating that ‘among the hundred who daily consult opticians, a very large proportion are unwilling to obtain a skilled opinion, that is, the opinion of an oculist due to cost’.^58^ The writer’s comment highlights the influence that potential customers or patients could have in limiting medical authority or jurisdiction. Indeed, the public’s preference to visit an optician encouraged the writer to concede total responsibility over vision testing and vision aid dispensing. While the writer, as an ‘oculist’, considered his own opinion as ‘skilled’, he listed the simple cases of refraction that were the opticians’ ‘domain’ and that would ensure ‘the best for their clients’.^59^ Medical practitioners also continued to acknowledge their own lack of appropriate training; ophthalmologists stood on the margins of a medical profession that focused on supporting a systemic, generalised form of medical training that did not encompass specialist training in vision testing.^60^ In May 1896 the journal discussed a recent report in the *Lancet*, which had stated that the ‘average practitioner’ would be beaten by the ‘practical optician’ in the accuracy of their vision testing and that few in the country or large towns ‘feel confident’ in their judgement.^61^ The journal marked this as a ‘step forward’ in the education of medical opinion. However, the acknowledgement of an optician’s skill relative to the average medical practitioner did not solve the problem of opticians needing to recognise disease or the status of medical training. In October 1896, an optician under the name ‘A Celtic Refractionist’ discussed how the existence of a ‘wretched jealousy’ on both sides prevented a positive working relationship and referral system being developed.^62^

Internal challenges and discrepancies further exaggerated external tensions and hindered *The Optician*’s ability to initially establish a standardised and cohesive community in the first half of the 1890s. Not all opticians had a desire, or the ambition, to upskill or professionalise in the way that *The Optician* promoted. Indeed, a significant proportion of opticians remained unwilling to professionalise because of its association with the delivery of a more complex vision test. This reluctance was based on the individual’s skill, or lack of it. The discrepancy in the favoured role of opticians was revealed in a months’ worth of correspondence between 21 February 1895 and 21 March 1895 under the heading ‘Opticians Should be Up and Doing’. This correspondence was triggered by ‘An Outsider’ who argued that opticians should form a solid body similar to dentists and ‘qualify in refraction and diagnose the existence of disease, but… not practice surgery or medicine’.^63^ However, the response from ‘An Insider’ in the next issue argued that certification was a ‘sheer waste of time’ and that even a ‘moderate standard of efficiency’ would result in the majority of opticians being ‘swept out of existence’.^64^ The correspondent considered there to be no comparison between opticians and dentists and the purpose of the optician was to let ‘the oculist do the hard work and fill the prescription myself (or rather let me manufacturer do it for me)’.^65^ Correspondence from the insider and outsider revealed that, on the one hand, there was a body of ‘scientific opticians’ pushing for change and the qualification of vision testing and, on the other hand, there were opticians who remained more aligned to the traditional techniques of simply selling optical wares. Indeed, in the next issue ‘an insider who intends to remain inside’ mocked ‘An Insider’’s resistance to change.^66^ The correspondent believed that ‘An Insider’’s approach would end in ‘poverty’; a belief also held by other opticians who saw a qualification in refraction as essential for surviving.^67^ Despite these criticisms, ‘An Insider’ responded by insisting that ‘there are not half-a-dozen opticians in this country who could pass any crucial examination in eye refraction or advanced theoretical or practical optics’.^68^

Certain opticians’ reluctance to proclaim vision testing expertise was based on the complexity of vision testing and the different skillset it required. The increasing sophistication of the vision test and task of fitting vision aids to the face, regardless of its commercial consequences, became a key argument for opticians that believed undertaking vision testing was not the best way to gain professional standing. On 4 June 1896, *The Optician* questioned that ‘if a house be divided against itself, how shall it stand?’ because of opposition that had sprung up from within the trade itself.^69^*The Optician* was even more concerned about this latest form of opposition because it was from someone of ‘high standing’, W.A. Dixey, a London optician whose business had served successive monarchs in the nineteenth century. Dixey had written to the *Lancet* to defend the practice of opticians. However, in doing so, he defined opticians’ specialised status through scientific knowledge of optics and not from medical knowledge of the eye. This conclusion was based on his belief that ‘the great increase during the last thirty years in the knowledge of ocular refraction and the therapeutic use of spectacles has lifted the whole matter into the professional sphere’.^70^ Dixey argued that the problem was not whether the optician or medical practitioner should test the sight, because a person should go ‘naturally’ to the medical practitioner, but because the organisation of vision testing and dispensing was ‘still in a state of transition’.^71^ In response, *The Optician* argued that some of the most able ‘refractionists’ were ophthalmic surgeons but that they failed to see why medical training, by default, could make medical practitioners superior.^72^ In contrast, Dixey justified his stance on the basis that medical training was useful and necessary for the prescription of spectacles as well as ‘ophthalmometry’. While Dixey acknowledged that an ‘orthodox course of surgical training’ was not the only possible way to change this knowledge, he warned the journal to not be blind to the state of change and ‘the lines along which affairs are developing’.^73^

Professional boundaries, regulation and monopoly were a concern of both opticians and medical practitioners in the early years of *The Optician.* However, opticians’ attempts to professionalise in response to the development of more sophisticated vision testing created conflict. The involvement of medical practitioners in dictating the process of vision testing fundamentally shaped the lines of development that Dixey was referring to. Indeed, Dixey’s arguments are indicative of certain opticians moving away from an association with vision testing in response to increasing medical involvement in the latter part of the 1890s. Yet, unlike other nascent professions, the comments of Dixey and others had limited overall impact and did not curb the overall trend towards opticians establishing their role in vision testing. A body of opticians were slowly moving towards establishing the role of the optometrist, a professional group able to test vision, prescribe lenses and refer cases of disease to the medical practitioner. Opticians, in defining their own distinctive skillset in this way, also shaped the extent to which they would be under medical practitioners’ control.

Dixey’s comment on the transitional stage of this process is crucial for understanding the tensions that existed both inside and outside the trade; medical knowledge and medical involvement in vision testing was new. While *The Optician’s* vision of a professional body of opticians was considered externally and internally by some to be challenging medical authority, ophthalmic surgeons’ justification of their authority over vision testing was problematised and weakened by the fact that vision testing in the medical curriculum and medical practice remained ill defined and hindered by the requisite to specialise. In response, opticians were able to create a rhetoric, which emphasised their optical scientific skill and their anatomical or physiological knowledge of the eye and made them uniquely qualified. *The Optician* was able to promote a professional identity based on their long association with vision aid dispensing. This challenged unqualified retailers, those who wished to continue specialising in scientific optics *and* medical practitioners, who were required to justify and define the legitimacy of their expertise.

## Certification and Examination, 1897–1904

By 1897 some of the initial aims of *The Optician* were being met. Early forms of certification were implemented by the BOA and then, a year later in 1898, the SMC followed suit. The competing institutions were not necessarily antagonistic in the early years. Members of the BOA were prominent in the SMC’s decision-making process and there was an initial hope that the two examining bodies would merge.^74^ Collectively, both institutions believed that a system of examinations would help to produce a standardised level of education that would be recognised by the public. The ability of examinations to aid in the production of a recognisable professional knowledge base is evident in the early reactions to the SMC’s re-involvement in *The Optician*. There were a number of calls for formal education to prepare applicants for the examinations and the SMC appointed a tutor at the Northampton Institute in Clerkenwell.^75^ To expand the scope of education beyond London there were practical training opportunities in different cities and the option to be educated via written correspondence.^76^ These initiatives were not isolated to the SMC, and the BOA similarly advertised its examinations alongside a scheme of tuition that would be held in London and Liverpool.^77^

The attendance and pass rate of the examinations in the early years of their implementation evidence optician’s claims of the role of certification in promoting a more cohesive, standardised, community of professionals. Examinations drew together opticians from a variety of locations. The BOA between 1898 and 1900 held their examinations in London and four other provincial locations: Dublin, Leeds, Liverpool and Manchester.^78^ Similarly, the provinces were ‘well represented’ in the London-based SMC examinations, including candidates from England, Scotland, Wales and Ireland.^79^ The broad geographical pool that candidates came from occurred in tandem with concerted efforts to improve and establish a national optical community. In marked contrast to the lone voices that attempted to instigate dentistry reform in the 1840s, opticians supported and made a concerted effort to implement the ideas that were promoted in *The Optician*.^80^ The London Optical Society was established in 1899 alongside more regional societies such as the Manchester Optical Society in 1898 and the West Riding Optical Society in 1900.^81^ Moreover, the number of successful candidates from the provinces passing the examinations outnumbered London candidates by March 1899 and is indicative of a nationwide change that involved both London and provincial opticians claiming qualifications and a professional area of expertise. Nevertheless, the number of successful candidates was significantly smaller than the candidates that entered. In December 1899 a report of the SMC’s November examinations stated that they were ‘very pleased’ that ‘so many’ as 61 candidates have been successful out of the 96 that sat the examination. The pass rate highlights, on the one hand, the standard of the BOA and SMC’s syllabuses. On the other hand, however, it demonstrates the range of candidates sitting the exams and continuing to be involved in vision aid prescribing.

Parallels can be drawn between the BOA and SMC examinations. A clause as the SMC implemented their examination, for example, allowed 46 members that had sat and passed one of the two higher level BOA examinations to receive SMC diplomas.^82^ Yet the BOA and SMC initially promoted two different kinds of expertise. The BOA was a newly established group of opticians that wished to protect the rights of opticians as vision testing specialists and improve their standing. The SMC was an independent livery company, which created a committee for the certification of opticians that included members of the BOA, opticians and ophthalmic surgeons. Neither institution wished to ‘antagonise’ the medical profession and believed a process of certification would help to foster favourable relationships.^83^ However, both the BOA and SMC offered an examination that encroached upon medical authority in different ways. As *The Optician* acknowledged, the SMC was a well-established body and had the potential to raise the status of the optician further. In contrast, the BOA was a relatively new organisation, but it did have more ambitious aims; indeed, it later became the College of Optometrists.

Comparing the first SMC examination with the BOA examination that took place a day later in November 1898 reveals the differences in the two institutions’ approach. Margaret Mitchell has argued that the BOA favoured the interests of opticians who specialised in vision testing, while the SMC fostered the interests of ophthalmic surgeons by limiting the optician’s skillset.^84^ In many respects this is true. The BOA’s written intermediate ‘dioptric’, and more advanced ‘ophthalmometric’, level examinations tested candidates on their understanding of refractive vision errors, as well as their ability to correct or identify them.^85^ Additionally, the practical examination required candidates to test a person’s vision under examination conditions.^86^ In comparison, the SMC examination did contain questions of a similar nature, including identifying a person’s refractive vision error and the appropriate lenses required. However, the SMC only examined candidates on vision testing as part of their theoretical written exam. The practical assessment was limited to measuring or fitting lenses to a person’s face based on the candidates’ ability to correctly read and interpret a prescription.^87^ The two institutions’ approach and focus were not markedly different; both expected the optician to have knowledge of optics, the principles of vision testing, the practicalities of measuring frames to the face, and the ability to follow prescriptions. However, what separated the two institutions in their initial years was the level of involvement, and control, of the ophthalmic surgeon in determining the appropriateness of the syllabus. The SMC examination would not include a vision testing examination until 1904.

Ophthalmic surgeons’ advice influenced the SMC’s decision to not include vision testing in its first examinations. Opticians and ophthalmic surgeons co-created the SMC examination, and its content therefore demonstrates how compromises could be made. Ophthalmic surgeon Dr George Lindsay Johnson vocally advocated opticians’ involvement in vision testing because of their ‘large experience’. Nevertheless, while Johnson argued that he would value an optician’s opinion before his own, he acknowledged that this view was not ‘shared by the medical profession in general’.^88^ The advice Johnson gave was therefore more conservative and he argued that they needed to consider the eye ‘merely as an optical instrument’. Johnson suggested that if opticians were examined on their knowledge of optics it would not encroach upon ‘the province of the qualified medical man’.^89^

As Johnson predicted, the syllabus did meet the approval of medical men. *The Lancet* argued that the examination syllabus would make the optician a ‘high instructed and competent man’, one who was able to recognise the cases that required a different level of knowledge so that ‘no loss on either side’ would be made and the patient would ultimately benefit.^90^*The British Medical Journal* also favourably reviewed the SMC scheme. However, initial tensions emerged from both sides relating to the question of appropriate conduct. Opticians in the *British Medical Journal* were presented as lying or posing as medical men because using the letters ‘F.S.M.C.’ or ‘B.O.A.’, when they passed the SMC or BOA examinations, could suggest to an ignorant public that they had received medical training.^91^ Medical practitioners focused on opticians’ advertising as part of their broader aversion to the growth of market capitalism in the sale of medical products in late Victorian Britain.^92^ Comment on other countries’ tougher regulations in the *British Medical Journal*, for example, show that vision testing debates were motivated by an opportunity to assert the superior status and practice of medicine.^93^ The SMC therefore conducted itself carefully in response to the heavy restrictions being placed on opticians by the medical profession, and the warnings that they gave about breaching the Medical Acts. The SMC had developed an Advertisement Sub-Committee for monitoring opticians’ advertisement and made successful examination candidates sign a legal agreement against the use of any form of drug or to advertise the diploma ‘in any way which would lead the public to infer that it conveys medical qualifications’.^94^

Despite early favourable reviews and the possibility of a working relationship, the realities of certification polarised the two professions. In March 1899, the Master of the SMC, while in favour of proper conduct, began to question the legitimacy of the medical profession’s intervention, arguing that the Medical Acts could not ‘prohibit’ the optician from learning their own business, which surgeons had only more recently become involved in.^95^ Similarly, medical practitioners increasingly questioned and recognised the legitimate competition of certificated opticians and therefore increased their retaliation. In May 1899 *The Optician* struggled to interpret the decision of medical practitioners to memorialise the General Medical Council on the subject of ‘the issue to persons not medically qualified, of certificates of proficiency in departments of medical practice’.^96^ Opticians were grouped with other professions such as midwifery, and the journal concluded that the memorial was wrongly informed and was an ‘injustice’ to the trade. Nevertheless, correspondence in the *British Medical Journal* focused on instances where ‘qualified’ opticians were wrong and had failed to provide a person with the correct prescription. In July 1902, for example, a correspondent argued that a chemist advertising as a ‘qualified optician’ had prescribed glasses that were ‘altogether wrong’ and that the diplomas were ‘responsible for the creation of a set of charlatans who, under the aegis of such diploma, mislead and impose on the public’.^97^*The British Medical Journal* in May 1903 argued that medical practitioners were not encroaching upon the rights of the opticians, but that opticians were failing to adhere to the guidelines that were initially formulated. It argued that opticians were not ‘experts’ and their attempts to establish a profession could not be compared to the development of the medical profession in the second half of the nineteenth century because they were not ‘professional’ in character:

An act of Parliament may do much to consolidate and strengthen a profession by trimming away from it the fringe of irregular practitioners, but it cannot give it those qualities which it does not already possess.^98^

The speed and intensity of medical practitioners’ actions are a reflection of opticians’ initial success in challenging the legitimacy of medical authority. Despite asserting optician’s inferior status, medical practitioners were unable to control opticians’ dispensing practices. Certification and qualification had given opticians greater cultural authority and had helped them to further establish themselves as vision testing *and* optical specialists. In 1903, for example, Johnson’s initial stance and advice on vision testing was changing. Johnson wrote into the *British Medical Journal* stating that he was stepping down as an examiner for the SMC until the matter of vision testing had been settled. Johnson argued that the optician’s trade ‘is not, and indeed cannot be, confined within the present limits of the SMC examination’.^99^ ‘Testing: The Eyesight of a Nation’ emerged as a debate in the *British Medical Journal’s* correspondence column in 1904 as the SMC was devising its new syllabus. A London optician, James Aitchison, argued that both opticians and ophthalmic surgeons were still adjusting to the recent changes in vision testing and the increasing involvement of ophthalmic surgeons. As a solution to this, Aitchison suggested that opticians needed to further their study of optics and refraction while ophthalmic surgeons focused on the study of eye diseases so that a favourable working partnership could be established. Aitchison concluded that ophthalmic surgeons’ greater engagement with opticians would allow them to see that the optician was a ‘sensible man’ and ‘frequently of considerable scientific attainments’.^100^ Aitchison’s statements highlight both the importance of character and the rhetoric of science in an attempt to gain the respect of the medical profession.

Aitchison emphasised the role of co-operation and the establishment of mutual interest as part of his claims. As a reputable optician, he argued that he was ‘frequently’ supplying glasses in refraction cases to the medical practitioner. A set of letters exchanged between London-based Johnson and Aitchison, survive and corroborate Aitchison’s claims.^101^ Their correspondence between 1898 and 1900 reveals how a working relationship between an optician and a medical practitioner could be established. The letters showed that Aitchison could prescribe glasses and send them to Johnson to double check, *and* Johnson could ask Aitchison to try and get a better prescription for the patient. In a letter from 7 June 1898, for example, Johnson discussed a patient’s eyesight and stated ‘I cannot get her vision better than 20/40 to 20/5 R & L… I wish you to see if you can improve her sight’.^102^ Looking at the relationship between Aitchison and Johnson highlights how the two individuals conceptualised their working relationship and what was considered important to uphold. Aitchison, for example, exchanged letters between 1901 and 1902 over concerns about opticians’ advertising. Additionally, the letters revealed how they favourably delineated their role based on two distinct skillsets. Correspondence from Johnson in the *British Medical Journal* in 1903 explicitly advocated the benefits of co-operation because of the lack of ophthalmic surgeons. Johnson saw ‘no possible solution except to recognise frankly the traditional right of the optician’.^103^ As the correspondence with Aitchison revealed, the ‘traditional right of the optician’ could be respected through a system of prescription and open dialogue between both parties. The trade directory entries for Aitchison are also revealing; they show that in 1895 he was listed as an ‘oculist optician’ but in 1899 had dropped the title of ‘oculist’ and was listed as an ‘optician’.^104^ In 1904, Aitchison was situating himself in the *British Medical Journal* firmly away from any association with medicine, and highlighting the increasing development and acknowledgement of specialised skillsets that would help to distinguish the two roles.^105^

Aitchison’s practical working relationship with Johnson may have been atypical. Indeed, Johnson frequently acknowledged the level of hostility he faced from medical practitioners because he supported opticians.^106^ This is also reflected in a response to Aitchison’s initial proposals in the *British Medical Journal.* One medical practitioner disregarded an optician’s skillset and reduced their occupational standing to a trade by arguing that ‘of all medical impositions on the public that of the *shopkeeper* deciding upon glasses is probably the most pernicious’.^107^ However, collaboration increasingly became a necessity. As argued by Johnson and acknowledged by ophthalmic surgeons in the early 1890s, the limited number of qualified ophthalmic surgeons meant that medically trained personnel were physically unable to deal with the size of the country’s optical requirements and new visual demands.^108^ Additionally, F. Dugon, another practitioner writing in the *British Medical Journal*, argued that individuals could choose not to attend an ophthalmic surgeon if they could not afford the fee or did ‘not wish to go to hospital for errors of refraction’.

Dugon’s correspondence argued that medical practitioners were unable to fully establish their position as a cultural authority on vision testing and that they did not have capacity to deal with the number of cases. As a result of this situation, Dugon argued that his collaboration with Aitchison had been positive and that ‘refraction is corrected perfectly by the competent optician’.^109^ Indeed, Dugon suggested that a number of medical practitioners had adopted this practice.^110^ In response a practitioner under the pseudonym ‘Oculus’ argued that the weakness in the system was the fees and cost of obtaining an ophthalmic surgeon’s ‘authoritative opinion’.^111^ However, ‘Oculus’ had missed the point that Dugon had made. The public at the start of the twentieth century did not necessarily see refractive vision errors and the use of spectacles as a medical problem that should be treated in a hospital setting. Indeed, *The Optician* also commented on the public’s inability to recognise the importance of suitable lenses, arguing in 1898 that changes in vision testing had not been yet acknowledged by the general public and that they were indifferent to the qualifications of the dispenser:

The ordinary individual (or at least *90 per cent of the public*, as I am able to glean from my experience in the trade) at the present time proceeds to buy a pair of glasses much in the same manner as he would do a pound of tea; and he is seen more concerned as to the quality of the tea than the suitability of the glasses he may select. His chief endeavour is to buy glasses as cheaply as he possibly can, while he will buy good tea and recognise the necessity of paying a decent price for it.^112^

The optician of Aitchison’s standing, as a ‘competent’ retailer with an increasingly more defined and scientific qualification, was more aligned with the public’s view on how vision aids should be obtained than the medical practitioner. While the introduction of sight testing into the SMC scheme was discussed in the *British Medical Journal* in 1904, the medical profession was unsuccessful in their attempts to curb it. ‘The Medico-Political Committee of the British Medical Association’ disapproved of the SMC’s introduction of vision testing, but they were unable to advise the SMC as they had done at the outset in 1898 and prevent vision testing being part of the diploma.^113^

## Conclusion

By 1904 opticians had made a concerted effort to establish a professional identity and implement change. Opticians’ examining bodies had moved closer to vision testing, a formal system of education was being established and optical societies had been developed. Opticians had positioned themselves as experts in vision testing and scientific optics and used this to configure their own professional knowledge base. Medical opposition to opticians’ attempts to reform their trade was explicit across the 1890s and early twentieth century. Medical practitioners increasingly attempted to assert their position as vision testing specialists by criticising and de-valuing opticians’ training and their position as retailers. Medical practitioners utilised technology and drew upon both the language of character and science to justify their claims and argue that vision testing required objective tests by medically trained personnel. However, medical practitioners were not successful in their attempts to subjugate the opticians’ position as a dispenser of vision aids. Medical practitioners could not be indifferent to the role of a certified optician and the expansive spectacle retail market. Opticians were able to draw upon their own rhetoric of science, which combined knowledge of optics as well as the optical and physical condition of the eye.

While this study of opticians’ and medical practitioners’ conduct in the 1890s is very much one about professional control, it is also a study of forced collaboration.^114^ Studying opticians in the context of medical authority, medical specialisation and medical professionalisation in the nineteenth century reveals how the language of science could be used by another occupation or trade to successfully hinder and limit the expansion of medical jurisdiction. Whether opticians’, or medical practitioners’, training and education in vision testing and vision aid dispensing actually improved in this period has not been the focus of this article. Regardless of any practical change, opticians were able to effectively construct a professional identity based on rhetoric because they were adopting and adapting the techniques being used by the medical profession to further their position. While ophthalmologists’ attempts to curb opticians’ efforts continued in earnest well into the early twentieth century, they, ultimately, were unable to prevent the passing of the Optician’s Act in 1958 and a body of opticians establishing their role as optometrists and gaining a professional register. It highlights how inter-professional debate both promoted and hindered the medicalisation of vision aid dispensing. Opticians were drawing upon medical knowledge and language to justify their position, which expanded the use of medical definitions in explaining the refractive condition of the eye. However, opticians also hindered the medicalisation of vision aid dispensing by configuring an area of expertise that was situated outside of medical knowledge and medical training and drew heavily on scientific, optical and marketing skill. The newness of vision testing and medical involvement, as well as the precarity of the ophthalmic surgeon’s position as a specialist outside of conventional medical teaching, weakened medical practitioner’s claims for authority and provided a space for opticians to establish and re-shape their position as vision testing experts. The period 1891–1904 was crucial for preventing the expansion of medical jurisdiction in response to the growing medical understanding and involvement in diagnosing and treating refractive vision errors. The actions of opticians in this period limited the extent of medicalisation and are subsequently able to show how, similar to other allied professions, medical practitioners were unable to control, and had to grudgingly accept, a partnership with a trade that drew upon a credible, and legitimate, rhetoric of scientific knowledge.

## Footnotes

1 ‘The Spectacle Makers’ Company’, *The Optician*, 21 April 1898, 210.

2 See, for example, William White Cooper, ‘On Myopia and Presbyopia’, *Provincial Medical Surgical Journal*, 23 July 1845, 470.

3 Chris Otter, *The Victorian Eye: A Political History of Light and Vision in Britain, 1800-1910* (Chicago; London: Chicago University Press, 2008), 52–54; Rosemarie Garland-Thompson, *Staring: How We Look* (Oxford: Oxford University Press, 2009), 26; Kate Flint, *The Victorians and the Visual Imagination* (Cambridge: Cambridge University Press, 2000), 1, 4, 5, 8.

4 Peter John Brownlee, *The Commerce of Vision: Optical Culture and Perception in Antebellum America* (Pennsylvania: University of Pennsylvania Press, 2018).

5 Jonathan Crary, *Techniques of the Observer: On Vision and Modernity in the Nineteenth Century* (Massachusetts: MIT Press, 1994), 1–24; Caroline Weaver, ‘Eyesight and Governance in Britain: Bureaucracy and the Senses in the 1920s’, *Social History of Medicine*, 2014, 27.2, 241–59, Caroline A. Jones, *Eyesight Alone: Clement Greenberg’s Modernism and the Bureaucratization of the Senses* (Chicago: University of Chicago Press, 2005); Jordanna Bailkin, ‘Colour Problems: Work, Pathology and Perception in Modern Britain’, *International Labor and Working-Class History*, 2005, 68, 93–111; for America, see Rosemary Stevens, *Medicine and the Public Interest: A History of Specialisation* (Berkley; Los Angeles; London: University of California Press, 1998), 104.

6 Caroline Weaver, ‘Eyesight and Governance in Britain: Bureaucracy and the Senses in the 1920s’, *Social History of Medicine*, 2014, 27.2, 241–59.

7 George Weisz, ‘The Emergence of Medical Specialisation in the Nineteenth Century’, *Bulletin for the History of Medicine*, 2003, 77, 536–75; George Weisz, *Divide and Conquer: A Comparative History of Medical Specialisation* (New York: Oxford University Press, 2006); George Rosen, *The Specialisation of Medicine with Particular Reference to Ophthalmology* (New York: Froben Press, 1944); Stephen Casper, *The Neurologists: A History of a Medical Specialty in Modern Britain, c.1789-2000* (Manchester: Manchester University Press, 2016), 60–64.

8 Graeme Gooday and Karen Sayer, *Managing the Experiences of Hearing Loss in Britain, 1830-1930* (Basingstoke: Palgrave Macmillan, 2017), 51.

9 Parallels can be drawn to audiometry. See, for example, Jaipreet Virdi-Dhesi’s ‘Curtis’s Cephaloscope: Deafness and the Making of Surgical Authority in London, 1816-1845’, *Bulletin of the History of Medicine*, 2013, 87.3, 347–77; Jaipreet Virdi and Coreen McGuire, ‘Phyllis M. Tookey Kerridge and the Science of Audiometric Standardization in Britain’, *British Journal for the History of Science*, 2018, 51.1, 123–46.

10 See, for example, N. David Richards, ‘Dentistry in England in the 1840s: The First Indications of a Movement Towards Professionalisation’, *Medical History*, 1968, 12, 137–52; Eric G. Forbes, ‘The Professionalisation of Dentistry in the United Kingdom’, *Medical History*, 1985, 29, 169–81; S. W. F. Holloway, ‘Producing Experts, Constructing Expertise: The School of Pharmacy of the Pharmaceutical Society of Great Britain, 1842-1896’, in Vivian Nutton and Roy Porter, eds, *The History of Medical Education in Britain* (Amsterdam: Rodopi, 1995), 116–40; Daniel J. Malleck, ‘Professionalism and the Boundaries of Control: Pharmacists, Physicians and Dangerous Substances in Canada, 1840-1908’, *Medical History*, 2004, 48.2, 175–98; Geoffrey Stuart Taylor and Malcolm Nicolson, ‘The Emergence of Orthodontics as a Speciality in Britain: The Role of the British Society for the Study of Orthodontics’, *Medical History*, 2007, 51, 379–98; Christine E. Hallett, ‘Nursing, 1830-1920: Forging a Profession’, in Anne Borsay and Billie Hunter, eds, *Nursing and Midwifery in Britain since 1700* (Basingstoke: Palgrave Macmillan, 2012), 46–73.

11 See particularly, Malleck, ‘Pharmacists, Physicians and Dangerous Substances’, 177–78 for an overview of his argument.

12 Jacqueline Jenkinson, ‘A “Crutch to Assist in Gaining an Honest Living”: Dispensary Shopkeeping by Scottish General Practitioners and the Responses of the British Medical Elite, ca 1852-1911’, *Bulletin of the History of Medicine*, 2012, 86.1, 1–36; Jacqueline Jenkinson, ‘More “Marginal Men”: A Prosopography of Scottish Shop-keeping Doctors in the Late Nineteenth and Early Twentieth Centuries’, *Social History of Medicine*, 2016, 29.1, 89–111.

13 S. E. D. Shortt, ‘Physicians, Science and Status: Issues in the Professionalisation of Anglo-American Medicine in the Nineteenth Century’, *Medical History*, 1983, 27, 51–68, see especially 62–63.

14 Richard Champness, *A Short History of the Worshipful Company of Spectacle Makers up to the Beginning of the Twentieth Century* (London: Apothecaries Hall, 1952); Margaret Mitchell, *History of the British Optical Association, 1895-1978* (London: British Optical Association, 1982).

15 Peter Conrad, *The Medicalisation of Society: On the Transformations of Human Conditions into Treatable Disorders* (Baltimore: The John Hopkins University Press, 2007), 9.

16 This practice can be traced to ophthalmologists’ medical books as early as William White Cooper’s *On Near Sight, Aged Sight, Impaired Vision and the Means of Assisting Sight*, 2nd edn (London: John Churchill, 1853), opticians’ advertised links with ophthalmic institutions and in popular descriptions, for example, ‘The Ophthalmic Institution and What I saw There’, *Aberdeen Weekly Journal*, 14 December 1894 (unpaginated). Although the utility of spectacles could still be debated see, for example, cases of complaint at Moorfields Eye Hospital. London Metropolitan Archive, A/KE/B/01/04/004: ‘Complaint That There Was Little Profit in Supplying Spectacles’ Charities’, letters dated 22 May 1899 and 17 May 1900.

17 For a comparative study of the different professions in America, see David V. Shaver, Jr, ‘Opticianry, Optometry, and Ophthalmology: An Overview’, *Medical Care*, 1974, 12.9, 754–65.

18 Shortt, ‘Physicians, Science and Status’, 51–52; See also Richards, ‘Dentistry in England’, 237–38.

19 Andrew Abbott, *The System of Professions: An Essay on the Division of Expert Labour* (Chicago: The University of Chicago Press, 1988), 1–9.

20 Conrad, 9.

21 See, for example, Richards, ‘Dentistry in England’, 145–48.

22 Ian A. Burney, ‘Medicine in the Age of Reform’, in Arthur Burns and Joanna Innes, eds, *Rethinking the Age of Reform: Britain 1780-1850* (Cambridge: Cambridge University Press, 2003), 163–64; Michael Brown, ‘Medicine, Reform and the “End of Charity” in Early Nineteenth-Century England’, *The English Historical Review*, 2009, 124.511, 1353–88, 1379–80; Richards, ‘Dentistry in England’, 145–48.

23 ‘Introducing “The Optician”’, *The Optician*, 2 April 1891, 2.

24 See discussion of retailers and reputability: John Benson, ‘Drink, Death and Bankruptcy: Retailing and Respectability in Late Victorian and Edwardian England’, *Midland History*, 2007, 32.1, 128–40; John Stobart, Andrew Hann and Victoria Morgan, *Spaces of Consumption: Leisure and Shopping in the English Town, c.1680-1830* (London: Routledge, 2007); Laura Ugolini and John Benson, eds, *Retailing Beyond the Shop, c. 1400-1900* (Bradford: Emerald Group Publishing Ltd, 2010).

25 Helen Berry, ‘Polite Consumption: Shopping in Eighteenth-Century England’, *Transactions of the Royal Historical Society*, 2002, 12, 375–94; Jon Stobart, ‘Selling (Through) Politeness: Advertising Provincial Shops in Eighteenth-Century England’, *Cultural and Social History*, 2008, 5.3, 309–28.

26 See, for example, Liliane Hilaire-Perez and Christelle Rabier, ‘Self-Machinery? Steel Trusses and the Management of Ruptures in eighteenth-century Europe’, *Technology and Culture*, 2013, 54, 460–502; David Turner and Alun Withey, ‘Technologies of the Body: Polite Consumption and the Correction of Deformity in Eighteenth-Century England’, *History: The Journal of the Historical Association*, 2014, 99.338, 775–96.

27 ‘The Certificated Optician’, *The Optician*, 20 August 1891, 325–26.

28 Ivan Waddington, ‘The Movement Towards the Professionalisation of Medicine’, *British Medical Journal*, 1990, 301.6754, 690; Irvine Loudon, ‘Medical Practitioners 1750-1850, and the Period of Medical Reform in Britain’ in Andrew Wear, ed, *Medicine in Society* (Cambridge: Cambridge University Press, 1992), 230; Malleck, ‘Pharmacists, Physicians and Dangerous Substances’, 184; Forbes, ‘Professionalisation of Dentistry’, 170–74; Richards, ‘Dentistry in England’, 138; Hallett, ‘Nursing’, 62–63, 66.

29 ‘The Voice of the Trade: An Institute of Qualified Opticians’, *The Optician*, 21 September 1893, 861.

30
*Ibid*.

31 ‘The Voice of the Trade: The Certified Optician’, *The Optician*, 1 October 1891, 30.

32 ‘The Voice of the Trade: Technical Lectures for Opticians’, *The Optician*, 31 March 1892, 28.

33 See, for example, the issues of 27 December 1894 and 27 January 1895.

34 Forbes, ‘Professionalisation of Dentistry’, 174.

35 See, for example, ‘The Ophthalmoscope’, *The Optician*, 8 October 1891, 38; ‘The Ophthalmoscope and its Uses’, ‘New Instrument for Quickly Determining Refractive Errors of the Eye’, *The Optician*, 16 July 1892, 208–10; *The Optician*, 13 July 1893, 710–12.

36 See, for example, ‘Testing and Astigmatism II’, *The Optician*, 28 January 1892, 292–93; ‘Determination of the Refractive Index’, *The Optician*, 8 September 1892, 409–10.

37 ‘The Optical Dictionary’, *The Optician*, 16 November 1893, 78; See, for example, ‘Papers for Beginners’, *The Optician*, 27 June 1895, 95; ‘The Optician’s Library’, *The Optician*, 31 October 1895, 77–78.

38 ‘The Ophthalmoscope’, *The Optician*, 13 June 1895, 137–39; 20 June 1895, 147–48; 4 July 1895, 167–68; 11 July 1895, 177; 18 July 1895, 188.

39 See, for example, ‘Literature’, *The Optician*, 26 January 1893, 312.

40 See, for example, *The Optician*, 9 April 1891, 23; 10 January 1895, 244.

41 For an overview of the debates, see: ‘The Voice of the Trade’, *The Optician*, 23 April 1891, 62;‘The Voice of the Trade’, *The Optician*, 9 July 1891, 238.

42 ‘The Voice of the Trade’, *The Optician*, 18 June 1891, 190.

43 ‘The Spectacle Makers’ Company and Technical Training’, *The Optician*, 10 March 1892, 385–86.

44 ‘Our Recent Remarks about Opticians and Oculists’, *The Optician*, 24 March 1892, 418.

45 ‘The Voice of the Trade’, *The Optician*, 4 January 1894, 142.

46 ‘The Voice of the Trade’, *The Optician*, 18 January 1894, 152.

47 See, for example, ‘The Voice of the Trade’, *The Optician*, 25 January 1894, 171.

48 ‘The Voice of the Trade’, *The Optician*, 18 January 1894, 161.

49 ‘The Voice of the Trade’, *The Optician*, 25 January 1894, 171.

50
*Ibid*.

51 ‘Oculist or Optician’, *The Optician*, 31 January 1895, 280.

52 ‘Optical Specialists and Medical Quacks’, *The Optician*, 7 February 1895, 294.

53 ‘Opticians and the Medical Profession’, *The Optician*, 14 February 1895, 308.

54 ‘Optical Specialists and Medical Quacks’, 294; ‘Opticians and the Medical Profession’, 308.

55 See, for example, ‘Oculist or Optician’, 280; and this was acknowledged in an address to the section of ophthalmology at the meeting of the British Medical Association in 1896, which was quoted in *The Optician*, 30 April 1896, 91–92.

56 George A. Berry (Royal College of Physicians of Edinburgh), *Diseases of the Eye: A Practical Treatise for Students of Ophthalmology*, 1st edn (Edinburgh and London: Young J. Pentland, 1889); Gustavus Hartridge, *The Refraction of the Eye: A Manual for Students* (London: J & A Churchill, 1884), iii–iv.

57 William Lang, *The Methodical Examination of the* *Eye:* *Being* *Part 1 of a* *Guide the* *Practice of* *Ophthalmology for* *Students and* *Practitioners* (London: Longmans, Green & Co, 1895).

58 ‘Can Opticians Be Trusted To Correct Defects of Sight? By an Oculist’, *The Optician*, 26 March 1896, 14.

59
*Ibid*.

60 Casper, *The Neurologists*, 64.

61 ‘A Step Forward’, *The Optician*, 14 May 1896, 119–20.

62 ‘The Voice of the Trade’, *The Optician*, 22 October 1896, 79.

63 ‘The Voice of the Trade: Opticians Should be Up and Doing’, *The Optician*, 21 February 1895, 323.

64
*Ibid*., 337.

65
*Ibid*.

66 ‘The Voice of the Trade: Opticians Should be Up and Doing’, *The Optician*, 7 March 1895, 353.

67
*Ibid*.; ‘Organising a British Optical Association’, *The Optician*, 14 March 1895, 366; ‘The Voice of the Trade: Opticians Should be Up and Doing’, *The Optician*, 21 March 1895, 380.

68 ‘The Voice of the Trade: Opticians Should be Up and Doing’*, The Optician*, 21 March 1895, 380–81.

69 ‘A House Divided Against Itself?’, *The Optician*, 4 June 1896, 164.

70
*Ibid*.

71
*Ibid*.

72 ‘Opticians and Oculists’, *The Optician*, 11 June 1896, 176.

73
*Ibid*., 178.

74 Mitchell, *History of the British Optical Association*, 60; See also, for example, ‘The S.M.C. Preliminary Steps’, *The Optician*, 27 January 1898, 437.

75 ‘Spectacle Makers Company’, *The Optician*, 7 July 1898, 528.

76 See advertisements in *The Optician*, 8 September 1898, 795*;* 15 December 1898, 462.

77 See, for example, *The Optician*, 22 September 1899, 961.

78 See, for example, ‘The B.O.A. Examination’, *The Optician*, 17 February 1898, 492; ‘The British Optical Association’, 5 May 1898, 254, Advertisement, 22 September 1899, 961.

79 ‘The Spectacle Makers’ Company Optical Examination’, *The Optician*, 10 November 1898, 313.

80 Richards, ‘Dentistry in England’, 138.

81 For the formation of the London Optical Society, see *The Optician*, 8 June 1899, 60–70; See also mention of the Manchester Optical Society in *The Optician*, 13 October 1898, 208, 1 December 1898, 397, 22 June 1899, 520; The West Riding Optical Society in *The Optician*, 9 February 1900, 743–44.

82 ‘Some Statistics Concerning the Examination Scheme of the Worshipful Company of Spectacle Makers’, *The Optician*, 30 March 1899, 37.

83 ‘The Spectacle Makers’ Company—Diplomas for Opticians’, *The Optician*, 23 December 1897, 348; ‘Oculists and Opticians’, *The Optician*, 20 January 1898, 419.

84 Mitchell, *History of the British Optical Association*, 61.

85 ‘The British Optical Association—November Examinations’, *The Optician*, 10 November 1898, 320.

86 ‘Voice of the Trade’, *The Optician*, 30 June 1898, 506.

87 ‘The Spectacle Makers’ Company Optical Examination’, *The Optician*, 10 November 1898, 313–18.

88 ‘Spectacle Makers’ Company’, *The Optician*, 24 February 1898, 522.

89
*Ibid*.

90 ‘The Revised Syllabus’, *The Optician*, 2 February 1899, 644.

91 ‘The Yorkshire Optical Society’, *British Medical Journal*, 24 June 1899, 1547.

92 Takahiro Ueyama, *Health in the Marketplace: Professionalism, Therapeutic Desires, and Medical Commodification in Late-Victorian London* (California: SPSS, 2010), 57.

93 See, for example, the *British Medical Journal*’s discussion of France in ‘Medical News’ on 23 January 1892, 203; and America in ‘The Ophthalmometrician’, 1 July 1899, 21.

94 ‘The Diplomas for Opticians—Letter From the Master of the Spectacle Makers’ Company’, *The Optician*, 16 March 1899, 842, and the committee meeting of the Spectacle Makers’ Company on the recent examination, 4 May 1899, 294.

95 ‘The Diplomas for Opticians—Letter From the Master of the Spectacle Makers’ Company’, 842–44.

96 ‘A Plea for Peace’, *The Optician*, 11 May 1899, 323–24.

97
*British Medical Journal*, 12 July 1902, 156.

98 ‘The Worshipful Company of Spectacle Makers and Eyesight Testing’, *British Medical Journal*, 23 May 1903, 1216–17.

99 George Lindsay Johnson, ‘The Examination of the Spectacle Makers Company: Sight Testing by Opticians’, *British Medical Journal*, 11 July 1903, 109–10.

100
*Ibid*.

101 Boots Archives, DA15/43: Collection of Letters from Lindsay Johnson to James Aitchison, 1898–1906.

102
*Ibid*.

103 Johnson, ‘Sight Testing by Opticians’, 109–10.

104
*Post Office London Trades Directory* (1895) [Part 3: Commercial and Professional Trade Directory], 771; *Post Office London Directory* (1899) [Part 3: Commercial and Professional Trade Directory], 853.

105 James Aitchison, ‘Correspondence: Testing the Eyesight of a Nation’, 14 May 1904, 1170.

106 See, for example, Johnson, ‘Sight Testing by Opticians’, *British Medical Journal*, 11 July 1903, 109–10.

107 A. Ogier Ward, ‘Correspondence: Testing the Eyesight of a Nation’, *British Medical Journal*, 28 May 1904, 1287-1288.

108 Johnson, ‘Sight Testing by Opticians’, 109–10.

109 F. Dugon, ‘Correspondence: Testing the Eyesight of a Nation’, *British Medical Journal*, 4 June 1904, 1345.

110
*Ibid*.

111 Oculus, ‘Correspondence: Testing the Eyesight of a Nation’, *British Medical Journal*, 25 June 1904, 1517.

112 ‘Voice of the Trade: The Certification of Opticians’, *The Optician*, 7 April 1898, 144.

113 The Medico-Political Committee, ‘The Spectacle Makers’ Company’, *Supplement to the* *British Medical Journal*, 16 July 1904, 36.

114 This argument has been made by Ueyama in relation to patent medicines, 107.

## Notes

Dr Gemma Almond is an honorary research fellow at Swansea University with a particular interest in disability history, medical history, material culture and the technological measurement of the body. She is currently completing a monograph on spectacles in Victorian Britain with Manchester University Press, which focuses on exploring the medicalisation and normalisation of vision testing standards and spectacle wear.

